# Association between age and the presence and mortality of breast cancer synchronous brain metastases in the United States: A neglected SEER analysis

**DOI:** 10.3389/fpubh.2022.1000415

**Published:** 2022-09-23

**Authors:** Wenqiang Che, Yujiao Wang, Xiangyu Wang, Jun Lyu

**Affiliations:** ^1^Department of Neurosurgery, The First Affiliated Hospital of Jinan University, Guangzhou, China; ^2^Department of Clinical Research, The First Affiliated Hospital of Jinan University, Guangzhou, China; ^3^Department of Pathology, Shanxi Provincial People's Hospital, Taiyuan, China

**Keywords:** breast cancer, brain metastases (BMs), restricted cubic spline (RCS), linearity, non-linearity, prognosis, SEER

## Abstract

**Background:**

The extent of the relationship between age and the presence of breast cancer synchronous brain metastases (BCSBMs) and mortality has not yet been well-identified or sufficiently quantified. We aimed to examine the association of age with the presence of BCSBMs and all-cause and cancer-specific mortality outcomes using the SEER database.

**Methods:**

Age-associated risk of the presence and survival of BCSBMs were evaluated on a continuous scale (restricted cubic spline, RCS) with logistic or Cox regression models. The main endpoints were the presence of BCSBMs and all-cause mortality or cancer-specific mortality. Cox proportional hazards regression and competing risk models were used in survival analysis.

**Results:**

Among 374,132 adult breast cancer patients, 1,441 (0.38%) had BMs. The presence of BCSBMs displayed a U-shaped relationship with age, with the highest point of the curve occurring at the age of 62. In both the younger (age ≤ 61) and older (age ≥ 62) groups, the observed curve showed a nearly linear relationship between age and the presence of BCSBMs. The relationship between age and all-cause mortality (ASM) and cancer-specific mortality (CSM) was linear. Older age at diagnosis was associated with a higher risk of ASM (HR 1.019, 95% CI: 1.013–1.024, *p* < 0.001) and CSM (HR 1.016, 95% CI: 1.010–1.023, *p* < 0.001) in multivariable Cox models. Age (sHR 1.007, 95% CI 1–1.013, *p* = 0.049) was substantially related to a significantly increased risk of CSM in competing risk models.

**Conclusion:**

Age had a non-linear U-shaped relationship with the presence of BCSBMs and a linear relationship with BCSBMs mortality.

## Introduction

The second most frequent solid tumor that can metastasize to the central nervous system is breast cancer (BC) (1, 2). Brain metastases (BMs) are expected to occur in 30–50% of people with metastatic BC (3–5). The brain microenvironment is vastly different from that of extracranial lesions, with its distinct cell types, architectural features, metabolic restrictions, and immunological milieu, which influence the metastatic process and treatment responses (6, 7). According to many important studies, HER2-positive disease, the existence of more than two metastatic sites at BC diagnosis, HR negative, and a more advanced stage of the original tumor were all linked to a greater risk of breast cancer BMs (4, 8, 9).

Despite this compelling evidence, there is less evidence about the associations between age and the presence of breast cancer synchronous brain metastases (BCSBMs). Studies on this topic found different results, with some studies showing that breast cancer BMs occurred more frequently among younger women (10, 11), one study considering that age had no impact on the presence of breast cancer BMs (12), some studies suggesting that patients with older age had greater odds of having breast cancer BMs (5, 9), and some studies regarding advanced age as a risk factor for the presence of BMs (13, 14). There are currently no reliable population-based estimations of the relationship between age and the occurrence of breast cancer BMs.

Prior studies establish that BMs confer a life-threatening prognosis for female BC patients (15–18). Diagnosed with BMs represents an independent risk factor for shorter survival time in a large cohort retrospective study, and it has been estimated that there is a 58% rise in the risk of death from all causes (15). Breast cancer patients previously had a median survival period of 3–6 months from the time their BMs were discovered (17). Another prospective study found that women with brain metastases have a median survival of 26.3 months, compared to 44.6 months for women who do not suffer from brain metastases (19). Furthermore, BMs have been shown to be a reliable predictor of bone metastases in patients with infiltrating duct carcinoma of the breast, which is associated with a worsening prognosis (20).

Age is a significant determinant in cancer incidence and survival, and it is also a considerable factor in BM survival (13, 14, 21). Studies have shown that increasing age has generally been associated with poorer survival of BMs (12–14). There were, however, some inconsistent results (11, 22). Furthermore, there is no credible data on the impact of age on the prognosis of breast cancer patients with BMs.

We aimed to examine in detail the association of age with the presence of synchronous BMs and all-cause and cancer-specific mortality outcomes at diagnosis of BC using a large, multicenter, contemporary, population-based cohort in the United States from the Surveillance, Epidemiology, and End Results (SEER) database.

## Methods

### Data sources and study population

We conducted a cohort study using data from the SEER program, which contains demographic, illness, and treatment-related information for 34.6 percent of cancer patients in the United States at the time of primary malignancy diagnosis (23). For patients diagnosed between 2010 and 2016, information about the presence or absence of brain metastases at the time of the initial systemic malignancy diagnosis was available. We examined 388,413 individuals aged 18 and above who were diagnosed with primary, invasive breast cancer between January 1, 2011, and December 31, 2016, in the SEER database.

We excluded individuals who were male (3 016 individuals), lacked data for education and household income (64 individuals), were diagnosed with carcinoma *in situ* (561 individuals), lacked information on bone metastases (9 380 individuals), liver metastases (487 individuals), lung metastases (443 individuals), and brain metastases (214 individuals). We also excluded cases if survival time was unknown (26 individuals). The final analytical cohort for the association of age and the presence of BCSBM included 374 132 participants. Of these, a total of 1,441 individuals were identified as having brain metastases. In addition, we excluded 6 participants whose diagnosis was based on an autopsy or death certificate, as well as one participant of undetermined race, leaving 1,434 participants eligible for survival analysis. Our institutional review board granted an exemption for this study since it is a secondary analysis of existing data (SEER).

### Covariates

Demographic variables included patients' age, sex, median household income, high school education percentage, year of diagnosis, race/ethnicity, registry region, marital status, and insurance status were reported by the SEER program. Clinical covariates such as tumor site, histological subtypes, T-stage, lymph nodal positive rate (LNPRate), and metastases in other organs (bone, liver, lung, and brain) were also included in the research. All the above variables were treated as factors, with the exception of age, household income, and education proportion, which were treated as continuous variables and given as mean and standard deviation (SD). The presence or absence of other organ metastases was confirmed before the start of treatment.

### Endpoints

In the logistic regression models, the primary endpoint was the presence of BMs at diagnosis. In Cox proportional hazard models, the primary endpoints were all-cause mortality (ASM) and cancer-specific mortality (CSM) based on the International Classification of Diseases, 10th revision (ICD-10) code recorded as the underlying cause of death. In the competing risk models that were analyzed using proportional subdistribution hazards models, the primary endpoint was cancer-specific mortality, while other causes of mortality were the competing risk (24, 25). The months to the event were calculated from the time of diagnosis to the end of follow-up or death.

### Statistical analysis

#### The presence of BMs

Independent factors in demographic variables and clinical covariates were used to determine whether independent factors were associated with the presence of BMs at diagnosis. The associations between age and the presence of BMs at diagnosis were evaluated on a continuous scale with restricted cubic spline curves (RCSs) based on logistic regression models with 4 knots at the 5th, 45th, 65th, and 90th percentiles of age (26). The spline model was adjusted for variables that were found to have significance in univariable logistic analysis (*p* ≤ 0.05). Then, sensitivity analyses were carried out to see if the findings were reliable.

Based on the cut-off value from the result of RCS, we divided the cohort into two age groups. The mean and SD were calculated for continuous variables, and the proportion was calculated for categorical variables in each age group. The *t*-test or chi-square test was used to calculate statistical differences for continuous and categorical variables. As the associations between age and the presence of BMs were approximately linear below and above the cut-off value, we additionally used multivariable logistic regression models to calculate the odds ratio (OR) and 95% confidence interval (CI).

#### The survival of BCSBMs

The hazard ratios of mortality were calculated using univariable and multivariable Cox proportional hazards regression models adjusted for possible confounders (27). We used restricted cubic spline models fitted to Cox proportional hazards models with 4 knots at the 5th, 45th, 65th, and 90th percentiles of age (26). ASM and CSM spline models were further adjusted for significant variables in ASM and CSM univariable Cox regression models, respectively.

A competing mortality risk regression analysis on cumulative incidence functions was conducted using Fine and Gray models to better estimate breast CSM and better account for the high rate of competing events (28, 29). The researchers calculated unadjusted and adjusted subdistribution hazard ratios (sHR) with 95% CI. The Cumulative Incidence Function (CIF) allows for estimating the incidence of CSM while accounting for competing risk.

Statistical analyses were conducted using R language program version 4.0.3 released on 10-10-2020 and STATA software version 14 (StataCorp). The 2-tailed α values of <0.05 were considered statistically significant.

## Results

Of data from 374 132 adult patients from the SEER database in the United States, Group A comprised 182,033 (48.6%) patients under the age of 61 (≤ 61), with a mean (SD) age of 50.49 (7.77), whereas Group B (age ≥ 62 years old) contained 192,099 (51.35 %) patients with a mean (SD) age of 72.60 (7.96). Among the entire cohort, 1,441 patients were diagnosed with BMs, accounting for 0.38% of the entire study population. Groups A and B had 775 (53.78%) and 666 (46.22%) BCSBMs, respectively, with the incidence proportion of BMs in Groups A and B being 0.43 percent and 0.35 percent. Tables 1, **3**, **4** show the baseline characteristics of the entire cohort and the BMs cohort.

**Table 1:** Baseline characteristics of study population.

**Variables**	**Categories**	**Patients with cancer (any stage): *N* (%)**	Group A*N (%)	Group B*N (%)	**Patients with brain metastases at diagnosis: *N* (%)**	**Incidence proportion of brain metastases**
Age*	Continuous	61.84 (13.56)	50.49 (7.77)	72.60 (7.96)	-	-
Year	2011	59,276 (15.84%)	29,711 (50.12)	29,565 (49.88)	230 (15.96)	0.39%
	2012	60,510 (16.17%)	29,901 (49.41)	30,609 (50.59)	206 (14.3)	0.34%
	2013	61,897 (16.54%)	30,227 (48.83)	31,670 (51.17)	258 (17.9)	0.42%
	2014	62,904 (16.81%)	30,597 (48.64)	32,307 (51.36)	255 (17.7)	0.41%
	2015	64,702 (17.29%)	31,076 (48.03)	33,626 (51.97)	271 (18.81)	0.42%
	2016	64,843 (17.33%)	30,521 (47.07)	34,322 (52.93)	221 (15.34)	0.34%
Race	NHW	254,330 (67.98%)	111,033 (43.66)	143,297 (56.34)	882 (61.21)	0.35%
	NHB	41,359 (11.05%)	23,175 (56.03)	18,184 (43.97)	262 (18.18)	0.63%
	NHAI/AN	2,078 (0.56%)	1,154 (55.53)	924 (44.47)	8 (0.56)	0.38%
	NHAPI	31,764 (8.49%)	18,884 (59.45)	12,880 (40.55)	109 (7.56)	0.34%
	Hispanic	42,459 (11.35%)	26,711 (62.91)	15748 (37.09)	179 (12.42)	0.42%
	Others	2,142 (0.57%)	1,076 (50.23)	1,066 (49.77)	1 (0.07)	0.05%
Region	Northeast	61,753 (16.51%)	30,181 (48.87)	31,572 (51.13)	265 (18.39)	0.43%
	Midwest	32,796 (8.77%)	15,234 (46.45)	17,562 (53.55)	120 (8.33)	0.37%
	South	81,533 (21.79%)	39,795 (48.81)	41,738 (51.19)	381 (26.44)	0.47%
	West	198,050 (52.94%)	96,823 (48.89)	101,227 (51.11)	675 (46.84)	0.34%
Marital status	Married	200,221 (53.52%)	110,259 (55.07)	89,962 (44.93)	601 (41.71)	0.3%
	Others	173,911 (46.48%)	71,774 (41.27)	102,137 (58.73)	840 (58.29)	0.48%
Insurance status	Insured	316,864 (84.69%)	146,250 (46.16)	170,614 (53.84)	986 (68.42)	0.31%
	Others	57,268 (15.31%)	35,783 (62.48)	21,485 (37.52)	455 (31.58)	0.79%
Primary tumor site	Central	18,861 (5.04%)	7,856 (41.65)	11,005 (58.35)	69 (4.79)	0.37%
	Upper-inner	45,492 (12.16%)	21,983 (48.32)	23,509 (51.68)	70 (4.86)	0.15%
	Lower-inner	20,571 (5.5%)	9,354 (45.47)	11,217 (54.53)	40 (2.78)	0.19%
	Upper-outer	124,529 (33.28%)	62,423 (50.13)	62,106 (49.87)	285 (19.78)	0.23%
	Lower-outer	27,880 (7.45%)	13,788 (49.45)	14,092 (50.55)	58 (4.02)	0.21%
	Axillary tail	2,020 (0.54%)	1,069 (52.92)	951 (47.08)	17 (1.18)	0.84%
	Others	134,779 (36.02%)	65,560 (48.64)	69,219 (51.36)	902 (62.6)	0.67%
T-Stage	1	216,452 (57.85%)	98,866 (45.68)	117,586 (54.32)	183 (12.7)	0.08%
	2	107,688 (28.78%)	57,307 (53.22)	50,381 (46.78)	305 (21.17)	0.28%
	3	22,452 (6%)	13,227 (58.91)	9,225 (41.09)	172 (11.94)	0.77%
	4	15,481 (4.14%)	7,254 (46.86)	8,227 (53.14)	453 (31.44)	2.93%
	Others	12,059 (3.22%)	5,379 (44.61)	6,680 (55.39)	328 (22.76)	2.72%
LNPRate	0–20%	221,250 (59.14%)	106,721 (48.24)	114,529 (51.76)	37 (2.57)	0.02%
	21–40%	23,323 (6.23%)	13,757 (58.98)	9,566 (41.02)	18 (1.25)	0.08%
	41–60%	14,493 (3.87%)	7,976 (55.03)	6,517 (44.97)	9 (0.62)	0.06%
	61–80%	6,589 (1.76%)	3,623 (54.99)	2,966 (45.01)	13 (0.9)	0.2%
	81–100%	45,984 (12.29%)	26,599 (57.84)	19,385 (42.16)	121 (8.4)	0.26%
	Unexamined	50,291 (13.44%)	15,926 (31.67)	34,365 (68.33)	1,019 (70.71)	2.03%
	Others	12,202 (3.26%)	7,431 (60.9)	4,771 (39.1)	224 (15.54)	1.84%
Subtype	HR+/HER2–	256,413 (68.54%)	116,506 (45.44)	139,907 (54.56)	550 (38.17)	0.21%
	HR+/HER2+	37,293 (9.97%)	22,427 (60.14)	14,866 (39.86)	214 (14.85)	0.57%
	HR–/HER2+	15,779 (4.22%)	9,620 (60.97)	6,159 (39.03)	165 (11.45)	1.05%
	Triple-negative	38,558 (10.31%)	21,842 (56.65)	16,716 (43.35)	256 (17.77)	0.66%
	Unknown	26,089 (6.97%)	11,638 (44.61)	14,451 (55.39)	256 (17.77)	0.98%
Bone metastases	No	360,946 (96.48%)	175,577 (48.64)	185,369 (51.36)	522 (36.22)	0.14%
	Yes	13,186 (3.52%)	6,456 (48.96)	6,730 (51.04)	919 (63.78)	6.97%
Liver metastases	No	369,081 (98.65%)	179,181 (48.55)	189,900 (51.45)	957 (66.41)	0.26%
	Yes	5,051 (1.35%)	2,852 (56.46)	2,199 (43.54)	484 (33.59)	9.58%
Lung metastases	No	367,815 (98.31%)	179,264 (48.74)	188,551 (51.26)	759 (52.67)	0.21%
	Yes	6,317 (1.69%)	2,769 (43.83)	3,548 (56.17)	682 (47.33)	10.8%
Brain metastases	No	372,691 (99.61%)	181,258 (48.63)	191,433 (51.37)	-	-
	Yes	1,441 (0.39%)	775 (53.78)	666 (46.22)	-	-
Income*	Continuous	0.66 (0.17)	0.66 (0.17)	0.65 (0.17)	-	-
Education*	Continuous	13.66 (5.74)	13.72 (5.76)	13.61 (5.72)	-	-
Total	-	374,132 (100)	182,033 (48.65)	192,099 (51.35)	1,441 (100)	0.38%

Income^*^, median household income, increased by per $10 000 annual; Education^*^, high school education percent, increased by per 10%; NHW, Non-Hispanic White; NHB, Non-Hispanic Black; NHAI/AN, Non-Hispanic American Indian/Alaska Native; NHAPI, Non-Hispanic Asian or Pacific Islander. Continuous variables^*^ are given as mean (standard deviation). Group A^*^, age ≤ 61; Group B^*^, age ≥ 62.

### Age and the presence of BMs

**Figure 1:**
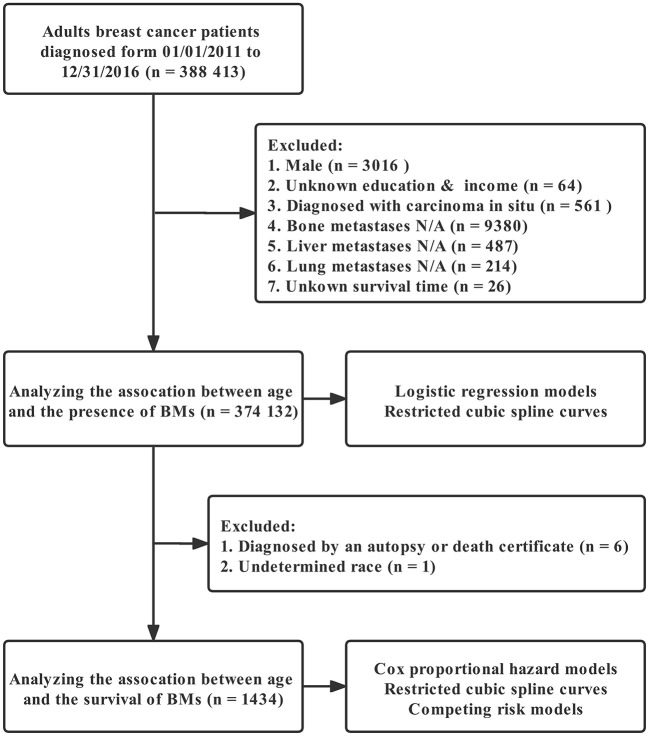
Analytical workflow.

**Figure 2:**
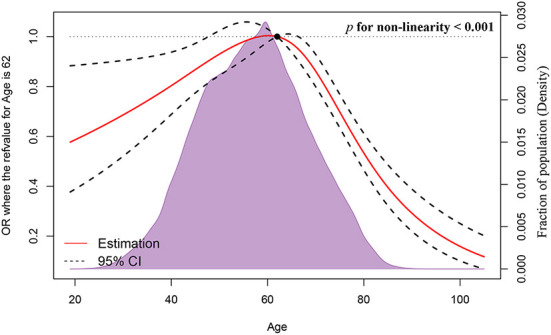
Association between age and the presence of BCSBMs using a restricted cubic spline regression model.

Figure 1 depicts the full analytical process. In univariable logistic regression, age (increased by per 1, odds ratio [OR], 0.99, 95%CI: 0.99–1, *p* < 0.001), non-Hispanic Black (NHB) (vs. non-Hispanic White [NHW], *p* < 0.001), Hispanic (vs. NHW, *p* = 0.017), West region (vs. Northeast, *p* = 0.001), other marital status (vs. married, *p* < 0.001), other insurance status (vs. insured, *p* < 0.001), tumor located in upper-inner or lower-inner or upper-outer or lower-outer or axillary tail of breast (vs. central, *p* < 0.001, *p* = 0.001, *p* < 0.001, *p* = 0.002, and *p* = 0.002, respectively), T-stage 2 or 3 or 4 (vs. 1, *p* < 0.001 for each one), LNPRate between 21 and 40% or between 41 and 60% or between 61 and 80% or between 81 and 100% (vs. 0–20%, *p* < 0.001 for each one), HR^+^/HER2^+^ or HR^−^/HER2^+^ or triple-negative subtype (vs. HR^+^/HER2^−^ subtype, *p* < 0.001 for each one), and metastasized to bone or liver or lung at diagnosis (vs. Not to, *p* < 0.001 for each one) were related to significantly greater odds of having BMs at diagnosis. Median household income (increased by per $10,000 annual, *p* < 0.001) was at lower risk of presence of BMs at diagnosed. Restricted cubic spline revealed a U-shaped relationship between age and the presence of BMs after controlling for the aforementioned possible confounders (Figure 2). The risk of having BMs increased rapidly until approximately the age of 62, after which it began to decline rapidly (P for non-linearity < 0.001). The results for sensitivity analyses are shown in Supplementary Figure 1. In multivariable logistic regression models, each year of age conferred a 1% (95% CI: 1–1.02, *p* = 0.036, p for non-linearity = 0.163) increase in the OR to develop BMs in Group A (age ≤ 61) and a 4% (95% CI: 0.95–0.97, *p* < 0.001, p for non-linearity = 0.067) decrease in the OR to develop BMs in Group B (age ≥ 62). Detailed data is shown in Table 2.

**Table 2:** Univariable and multivariable logistic regression models for the presence of breast cancer synchronous brain metastases.

**Variables**	**Categories**	**Univariable**		**Multivariable**		**Multivariable**		**Multivariable**	
				**Total**		Group A*		Group B*	
		**OR (95%CI)**	***p*-Value**	**OR (95%CI)**	***p*-Value**	**OR (95%CI)**	***p*-Value**	**OR (95%CI)**	***p*-Value**
Age	Continuous	0.99 (0.99, 1)	< 0.001	0.99 (0.98, 0.99)	< 0.001	1.01 (1, 1.02)	0.036	0.96 (0.95, 0.97)	< 0.001
Year	2011	Ref.		-	-	-	-	-	-
	2012	0.88 (0.73, 1.06)	0.172	-	-	-	-	-	-
	2013	1.07 (0.9, 1.28)	0.429	-	-	-	-	-	-
	2014	1.04 (0.87, 1.25)	0.63	-	-	-	-	-	-
	2015	1.08 (0.91, 1.29)	0.393	-	-	-	-	-	-
	2016	0.88 (0.73, 1.06)	0.168	-	-	-	-	-	-
Race	NHW	Ref.		Ref.		Ref.		Ref.	
	NHB	1.83 (1.6, 2.1)	< 0.001	0.98 (0.84, 1.15)	0.836	0.95 (0.77, 1.17)	0.632	1.04 (0.82, 1.32)	0.733
	NHAI/AN	1.11 (0.55, 2.23)	0.768	0.998 (0.475, 2.101)	0.997	1.04 (0.4, 2.7)	0.936	0.9 (0.27, 2.96)	0.86
	NHAPI	0.99 (0.81, 1.21)	0.917	0.97 (0.78, 1.2)	0.764	1.17 (0.9, 1.53)	0.239	0.63 (0.41, 0.96)	0.031
	Hispanic	1.22 (1.04, 1.43)	0.017	1.05 (0.88, 1.26)	0.581	1.02 (0.8, 1.29)	0.89	1.22 (0.91, 1.62)	0.186
	Others	0.13 (0.02, 0.95)	0.044	0.11 (0.01, 0.77)	0.026	0.2 (0.03, 1.48)	0.116	0 (0, 4.15*10,145)	0.942
Region	Northeast	Ref.		Ref.		Ref.		Ref.	
	Midwest	0.85 (0.69, 1.06)	0.147	1.08 (0.84, 1.37)	0.559	1.22 (0.85, 1.74)		0.995 (0.71, 1.39)	0.978
	South	1.09 (0.93, 1.27)	0.286	1.28 (1.05, 1.56)	0.014	1.5 (1.13, 1.99)		1.11 (0.84, 1.47)	0.469
	West	0.79 (0.69, 0.91)	0.001	1.0q (0.859, 1.179)	0.935	1.19 (0.95, 1.51)		0.86 (0.69, 1.08)	0.191
Marital status	Married	Ref.		Ref.		Ref.		Ref.	
	Others	1.61 (1.45, 1.79)	< 0.001	1.01 (0.894, 1.131)	0.922	0.95 (0.81, 1.12)	0.593	1.18 (0.99, 1.4)	0.064
Insurance status	Insured	Ref.		Ref.		Ref.		Ref.	
	Others	2.57 (2.3, 2.87)	< 0.001	1.22 (1.07, 1.39)	0.003	1.32 (1.12, 1.56)	0.001	1.03 (0.83, 1.27)	0.802
Primary tumor site	Central	Ref.		Ref.		Ref.		Ref.	
	Upper-inner	0.42 (0.3, 0.59)	< 0.001	0.97 (0.68, 1.37)	0.85	1.41 (0.85, 2.34)	0.19	0.7 (0.43, 1.15)	0.155
	Lower-inner	0.53 (0.36, 0.78)	0.001	1.04 (0.69, 1.57)	0.841	1.46 (0.82, 2.62)	0.201	0.79 (0.44, 1.42)	0.428
	Upper-outer	0.62 (0.48, 0.81)	< 0.001	1.08 (0.82, 1.43)	0.582	1.39 (0.91, 2.13)	0.126	0.89 (0.61, 1.3)	0.543
	Lower-outer	0.57 (0.4, 0.81)	0.002	1.03 (0.71, 1.49)	0.874	1.23 (0.71, 2.1)	0.461	0.91 (0.55, 1.5)	0.704
	Axillary tail	2.31 (1.36, 3.94)	0.002	2.07 (1.17, 3.65)	0.012	2.3 (0.99, 5.34)	0.054	2.06 (0.95, 4.46)	0.067
	Others	1.83 (1.44, 2.35)	< 0.001	1.37 (1.05, 1.77)	0.019	1.72 (1.15, 2.56)	0.009	1.16 (0.82, 1.63)	0.405
T-Stage	1	Ref.		Ref.		Ref.		Ref.	
	2	3.36 (2.79, 4.03)	< 0.001	1.33 (1.1, 1.62)	0.003	1.1 (0.85, 1.42)	0.487	1.74 (1.3, 2.32)	< 0.001
	3	9.12 (7.41, 11.24)	< 0.001	1.66 (1.32, 2.08)	< 0.001	1.12 (0.82, 1.54)	0.461	2.71 (1.94, 3.77)	< 0.001
	4	35.62 (29.98, 42.33)	< 0.001	1.97 (1.62, 2.41)	< 0.001	1.69 (1.3, 2.21)	< 0.001	2.27 (1.69, 3.07)	< 0.001
	Others	33.04 (27.55, 39.63)	< 0.001	2.47 (2.01, 3.03)	<0.001	2.06 (1.55, 2.73)	<0.001	3.02 (2.23, 4.09)	<0.001
LNPRate	0–20%	Ref.		Ref.		Ref.		Ref.	
	21–40%	4.62 (2.63, 8.11)	<0.001	3.45 (1.96, 6.08)	<0.001	4.9 (2.46, 9.75)	<0.001	1.81 (0.61, 5.41)	0.288
	41–60%	3.71 (1.79, 7.7)	<0.001	2.69 (1.3, 5.6)	0.008	3.98 (1.68, 9.46)	0.002	1.34 (0.31, 5.82)	0.698
	61–80%	11.82 (6.28, 22.24)	<0.001	6.61 (3.49, 12.53)	<0.001	8.94 (4.03, 19.85)	<0.001	4.42 (1.47, 13.29)	0.008
	81–100%	15.77 (10.91, 22.8)	<0.001	8.86 (6.09, 12.9)	<0.001	9.87 (5.97, 16.32)	<0.001	8.26 (4.69, 14.56)	<0.001
	Unexamined	123.64 (89.06, 171.67)	<0.001	27.52 (19.47, 38.89)	<0.001	30.85 (19.2, 49.57)	<0.001	26.98 (16.22, 44.88)	<0.001
	Others	111.81 (78.92, 158.39)	<0.001	20.66 (14.33, 29.77)	<0.001	21.97 (13.39, 36.06)	<0.001	22.01 (12.75, 38)	<0.001
Subtype	HR^+^/HER2^−^	Ref.		Ref.		Ref.		Ref.	
	HR^+^/HER2^+^	2.68 (2.29, 3.15)	<0.001	1.51 (1.27, 1.79)	<0.001	1.57 (1.26, 1.97)	<0.001	1.48 (1.13, 1.94)	0.004
	HR^−^/HER2^+^	4.92 (4.13, 5.85)	<0.001	2.48 (2.04, 3.02)	<0.001	2.58 (2.01, 3.33)	<0.001	2.43 (1.78, 3.31)	<0.001
	Triple-negative	3.11 (2.68, 3.61)	<0.001	2.68 (2.28, 3.15)	<0.001	2.99 (2.4, 3.72)	<0.001	2.45 (1.92, 3.12)	<0.001
	Unknown	4.61 (3.97, 5.35)	<0.001	1.47 (1.25, 1.74)	<0.001	1.65 (1.29, 2.11)	<0.001	1.39 (1.11, 1.75)	0.004
Bone metastases	No	Ref.		Ref.		Ref.		Ref.	
	Yes	51.73 (46.39, 57.68)	<0.001	5.2 (4.53, 5.96)	<0.001	6.33 (5.2, 7.7)	<0.001	3.94 (3.25, 4.78)	<0.001
	No	Ref.		Ref.		Ref.		Ref.	
	Yes	40.77 (36.4, 45.65)	<0.001	1.88 (1.64, 2.15)	<0.001	1.68 (1.4, 2.02)	<0.001	2.18 (1.78, 2.66)	<0.001
Lung metastases	No	Ref.		Ref.		Ref.		Ref.	
	Yes	58.53 (52.61, 65.12)	<0.001	4.31 (3.79, 4.89)	<0.001	3.95 (3.3, 4.71)	<0.001	4.44 (3.7, 5.34)	<0.001
Income*	Continuous	0.61 (0.45, 0.83)	<0.001	1.31 (0.89, 1.93)	0.175	1.28 (0.75, 2.19)	0.373	1.45 (0.82, 2.55)	0.202
Education*	Continuous	1 (1.99, 1)	0.724	-	-	-	-	-	-

Income^*^, median household income, increased by per $10 000 annual; Education^*^, high school education percent, increased by per 10%; NHW, Non-Hispanic White; NHB, Non-Hispanic Black; NHAI/AN, Non-Hispanic American Indian/Alaska Native; NHAPI, Non-Hispanic Asian or Pacific Islander. Group A^*^, age ≤ 61; Group B^*^, age ≥ 62.

### Age and mortality

**Figure 3:**
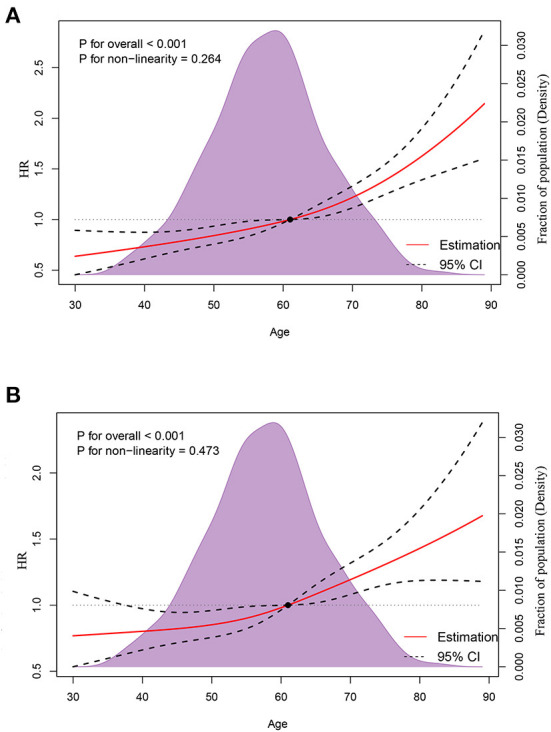
Association between age and all-cause mortality **(A)** and cancer-specific mortality **(B)** using restricted cubic spline regression models.

A median (interquartile range) of 16 (6–32) months was the time period of mortality ascertainment, corresponding to 1,042 deaths from all causes and 807 deaths caused by cancer. RCS indicated an ascending all-cause mortality risk with increasing age (Figure 3A, p for non-linearity = 0.264), controlled for median household income, race, region, marital status, tumor site, LNPRate, histological subtype, and metastasized to bone, liver, and lung. There was also an escalating cancer-specific mortality risk with increasing age (Figure 3B, p for non-linearity = 0.473), controlled for median household income, race, marital status, insurance status, T-stage, histological subtype, and metastasized to the liver and lung. Additionally, in multivariable Cox proportional-hazard models, the adjusted HRs of age for mortality due to all-cause and cancer-specific were HR = 1.019 (95% CI: 1.013–1.024, *p* < 0.001) and HR = 1.016 (95% CI: 1.010–1.023, *p* < 0.001), respectively. The corresponding detailed results of the unadjusted and adjusted Cox models are shown in Tables 3, 4.

**Table 3:** Distribution of breast cancer synchronous brain metastases all-cause mortality and hazard risk associated with various prognostic factors.

**Variables**	**Categories**	**Deaths *N* (%)**	**Univariable**		**Multivariable**	
			**HR (95%)**	***p*-Value**	**HR (95%)**	***p*-Value**
Age	Continuous	1,042 (100)	1.018 (1.013, 1.024)	<0.001	1.019 (1.013, 1.024)	<0.001
Year	2011	208 (19.96)	Ref.		Ref.	
	2012	178 (17.08)	0.957 (0.782, 1.170)	0.665		
	2013	210 (20.15)	0.948 (0.780, 1.153)	0.594		
	2014	199 (19.1)	1.077 (0.882, 1.314)	0.468		
	2015	170 (16.31)	0.997 (0.809, 1.229)	0.978		
	2016	77 (7.39)	0.895 (0.683, 1.173)	0.422		
Race	NHW	637 (61.13)	Ref.		Ref.	
	NHB	213 (20.44)	1.301 (1.113, 1.519)	0.001	1.266 (1.071, 1.496)	0.006
	NHAI/AN	5 (0.48)	0.603 (0.250, 1.453)	0.259	0.783 (0.322, 1.905)	0.590
	NHAPI	70 (6.72)	0.968 (0.756, 1.239)	0.793	1.180 (0.912, 1.527)	0.208
	Hispanic	117 (11.23)	0.859 (0.705, 1.046)	0.131	0.909 (0.739, 1.118)	0.366
Region	Northeast	194 (18.62)	Ref.		Ref.	
	Midwest	97 (9.31)	1.214 (0.951, 1.549)	0.119	1.008 (0.776, 1.310)	0.951
	South	293 (28.12)	0.956 (0.797, 1.146)	0.628	0.915 (0.733, 1.143)	0.434
	West	458 (43.95)	0.840 (0.710, 0.993)	0.042	0.927 (0.774, 1.110)	0.407
Marital status	Married	405 (38.87)	Ref.		Ref.	
	Others	637 (61.13)	1.350 (1.192, 1.530)	<0.001	1.211 (1.062, 1.380)	0.004
Insurance status	Insured	722 (69.29)	Ref.		Ref.	
	Others	320 (30.71)	1.026 (0.899, 1.170)	0.704		
Primary tumor site	Central	45 (4.32)	Ref.		Ref.	
	Upper-inner	57 (5.47)	1.593 (1.078, 2.356)	0.020	1.591 (1.071, 2.363)	0.022
	Lower-inner	26 (2.5)	0.892 (0.550, 1.446)	0.641	1.026 (0.629, 1.675)	0.917
	Upper-outer	188 (18.04)	1.113 (0.804, 1.541)	0.520	1.340 (0.963, 1.865)	0.082
	Lower-outer	41 (3.93)	1.255 (0.822, 1.917)	0.292	1.461 (0.952, 2.242)	0.083
	Axillary tail	14 (1.34)	1.495 (0.820, 2.725)	0.189	1.766 (0.957, 3.262)	0.069
	Others	671 (64.4)	1.324 (0.979, 1.791)	0.068	1.430 (1.052, 1.944)	0.022
T-Stage	1	131 (12.57)	Ref.		Ref.	
	2	203 (19.48)	0.968 (0.777, 1.207)	0.774		
	3	126 (12.09)	1.057 (0.828, 1.350)	0.655		
	4	340 (32.63)	1.190 (0.973, 1.456)	0.091		
	Others	242 (23.22)	1.137 (0.919, 1.407)	0.236		
LNPRate	0–20%	21 (2.02)	Ref.		Ref.	
	21–40%	11 (1.06)	0.775 (0.373, 1.607)	0.493	0.875 (0.419, 1.827)	0.722
	41–60%	5 (0.48)	0.694 (0.261, 1.841)	0.463	0.782 (0.293, 2.086)	0.624
	61–80%	10 (0.96)	1.087 (0.512, 2.309)	0.829	1.267 (0.594, 2.704)	0.541
	81–100%	83 (7.97)	1.177 (0.729,1.899)	0.506	1.208 (0.744,1.964)	0.445
	Unexamined	758 (72.74)	1.599 (1.036, 2.468)	0.034	1.505 (0.967, 2.343)	0.070
	Others	154 (14.78)	1.384 (0.877, 2.185)	0.163	1.423 (0.894, 2.262)	0.137
Subtype	HR+/HER2–	369 (35.41)	Ref.		Ref.	
	HR+/HER2+	129 (12.38)	0.766 (0.626, 0.936)	0.009	0.770 (0.627, 0.945)	0.012
	HR–/HER2+	112 (10.75)	1.090 (0.882, 1.346)	0.427	1.076 (0.865, 1.338)	0.513
	Triple-negative	215 (20.63)	2.020 (1.702, 2.398)	<0.001	2.111 (1.763, 2.529)	<0.001
	Unknown	217 (20.83)	1.986 (1.678, 2.351)	<0.001	1.882 (1.579, 2.242)	<0.001
Bone metastases	No	388 (37.24)	Ref.		Ref.	
	Yes	654 (62.76)	0.878 (0.775, 0.996)	0.043	0.899 (0.784, 1.030)	0.123
Liver metastases	No	672 (64.49)	Ref.		Ref.	
	Yes	370 (35.51)	1.372 (1.208, 1.559)	<0.001	1.543 (1.344, 1.770)	<0.001
Lung metastases	No	538 (51.63)	Ref.		Ref.	
	Yes	504 (48.37)	1.217 (1.077, 1.374)	0.002	1.119 (0.985, 1.273)	0.085
Income*	Continuous	1,042 (100)	0.570 (0.405, 0.800)	0.001	0.497 (0.324, 0.763)	0.001
Education*	Continuous	1,042 (100)	1.000 (0.990, 1.011)	0.971	-	-

Income^*^, median household income, increased by per $10 000 annual; Education^*^, high school education percent, increased by per 10%; NHW, Non-Hispanic White; NHB, Non-Hispanic Black; NHAI/AN, Non-Hispanic American Indian/Alaska Native; NHAPI, Non-Hispanic Asian or Pacific Islander.

**Table 4:** Distribution of breast cancer synchronous brain metastases cancer-specific mortality and hazard risk associated with various prognostic factors.

**Variables**	**Categories**	**Deaths *N* (%)**	**Univariable**		**Multivariable**	
			**HR (95%)**	***p*-Value**	**HR (95%)**	***p*-Value**
Age		807 (100%)	1.013 (1.007, 1.019)	<0.001	1.016 (1.010, 1.023)	<0.001
Year	2011	167 (20.69%)	Ref.		Ref.	
	2012	132 (16.36%)	0.886 (0.704, 1.11)	0.300	-	-
	2013	172 (21.31%)	0.972 (0.782, 1.207)	0.797	-	-
	2014	148 (18.34%)	0.996 (0.794, 1.249)	0.970	-	-
	2015	127 (15.74%)	0.923 (0.727, 1.171)	0.507	-	-
	2016	61 (7.56%)	0.889 (0.656 1.204)	0.447	-	-
Race	NHW	493 (61.09%)	Ref.		Ref.	
	NHB	165 (20.45%)	1.302 (1.091, 1.553)	0.003	1.153 (0.959, 1.387)	0.131
	NHAI/AN	5 (0.62%)	0.790 (0.327, 1.906)	0.599	1.094 (0.449, 2.665)	0.843
	NHAPI	55 (6.82%)	0.983 (0.743, 1.299)	0.901	1.071 (0.803, 1.427)	0.642
	Hispanic	89 (11.03%)	0.846 (0.675, 1.06)	0.147	0.843 (0.669, 1.063)	0.149
Region	Northeast	148 (18.34%)	Ref.		Ref.	
	Midwest	75 (9.29%)	1.231 (0.932, 1.625)	0.143	-	-
	South	233 (28.87%)	1.000 (0.814, 1.229)	0.999	-	-
	West	351 (43.49%)	0.847 (0.699, 1.026)	0.090	-	-
Marital status	Married	304 (37.67%)	Ref.		Ref.	
	Others	503 (62.33%)	1.418 (1.229, 1.635)	0.001	1.266 (1.090, 1.472)	0.002
Insurance status	Insured	536 (66.42%)	Ref.		Ref.	
	Others	271 (33.58%)	1.175 (1.015, 1.36)	0.030	1.165 (0.994, 1.365)	0.060
Primary tumor site	Central	36 (4.46%)	Ref.		Ref.	
	Upper-inner	43 (5.33%)	1.493 (0.958, 2.325)	0.076	-	-
	Lower-inner	16 (1.98%)	0.679 (0.376, 1.224)	0.197	-	-
	Upper-outer	149 (18.46%)	1.0994 (0.764, 1.582)	0.610	-	-
	Lower-outer	31 (3.84%)	1.181 (0.731, 1.910)	0.497	-	-
	Axillary tail	6 (0.74%)	0.783 (0.330, 1.859)	0.579	-	-
	Others	526 (65.18%)	1.289 (0.919, 1.806)	0.142	-	-
**T-Stage**	1	82 (10.16%)	Ref.		Ref.	
	2	156 (19.33%)	1.191 (0.911, 1.556)	0.202	1.234 (0.942, 1.618)	0.127
	3	102 (12.64%)	1.378 (1.030, 1.843)	0.031	1.340 (0.998, 1.801)	0.052
	4	287 (35.56%)	1.615 (1.263, 2.064)	<0.001	1.536 (1.197, 1.972)	0.001
	Others	180 (22.3%)	1.353 (1.042, 1.757)	0.023	1.331 (1.022, 1.733)	0.034
LNPRate	0–20%	19 (2.35%)	Ref.		Ref.	
	21–40%	10 (1.24%)	0.779 (0.362, 1.676)	0.522	-	-
	41–60%	5 (0.62%)	0.771 (0.288, 2.066)	0.605	-	-
	61–80%	8 (0.99%)	0.965 (0.422, 2.206)	0.933	-	-
	81–100%	64 (7.93%)	0.999 (0.598, 1.667)	0.995	-	-
	Unexamined	577 (71.5%)	1.334 (0.844, 2.109)	0.217	-	-
	Others	124 (15.37%)	1.230 (0.758, 1.993)	0.404	-	-
Subtype	HR+/HER2–	289 (35.81%)	Ref.		Ref.	
	HR+/HER2+	110 (13.63%)	0.836 (0.670, 1.041)	0.109	0.865 (0.693, 1.081)	0.202
	HR–/HER2+	90 (11.15%)	1.118 (0.883, 1.417)	0.355	1.072 (0.842, 1.364)	0.573
	Triple-negative	169 (20.94%)	2.014 (1.659, 2.444)	<0.001	2.185 (1.792, 2.665)	<0.001
	Unknown	149 (18.46%)	1.715 (1.406, 2.092)	<0.001	1.777 (1.448, 2.181)	<0.001
Bone metastases	No	286 (35.44%)	Ref.		Ref.	
	Yes	521 (64.56%)	0.952 (0.824, 1.100)	0.507	-	-
Liver metastases	No	506 (62.7%)	Ref.		Ref.	
	Yes	301 (37.3%)	1.480 (1.282, 1.707)	<0.001	1.616 (1.389, 1.880)	<0.001
Lung metastases	No	405 (50.19%)	Ref.		Ref.	
	Yes	402 (49.81%)	1.288 (1.122, 1.479)	<0.001	1.169 (1.012, 1.350)	0.033
Income*	Continuous	807 (100%)	0.403 (0.273, 0.596)	<0.001	0.408 (0.271, 0.615)	<0.001
Education*	Continuous	807 (100%)	1.006 (0.994, 1.018)	0.317	-	-

Income^*^, median household income, increased by per $10 000 annual; Education^*^, high school education percent, increased by per 10%; NHW, Non-Hispanic White; NHB, Non-Hispanic Black; NHAI/AN, Non-Hispanic American Indian/Alaska Native; NHAPI, Non-Hispanic Asian or Pacific Islander.

In addition, we evaluated CSM using a competing risk model. The CIF curves for the observed risk of CSM are shown in Supplementary Figure 2. Table 5 shows the results of multivariable competing-risk regression analyses predicting the time to CSM. Age (increased by per 1, sHR 1.007, 95% CI 1–1.013, *p* = 0.049), other marital status (vs. married, sHR 1.191, 95% CI 1.016–1.397, *p* < 0.001), other insurance status (vs. insured, sHR 1.221, 95% CI 1.034–1.442, *p* < 0.001), T-stage 3 (vs. 1, sHR 1.438, 95% CI 1.057–1.956, *p* = 0.021), T-stage 4 (vs. 1, sHR 1.612, 95% CI 1.242–2.091, *p* < 0.001), triple-negative subtype (vs. HR^+^/HER2^−^ subtype, sHR 1.693, 95% CI 1.374–2.086, *p* < 0.001), and metastasized to liver (vs. not to, sHR 1.367, 95% CI 1.163–1.607, *p* < 0.001) were significantly associated with an increased risk for CSM.

**Table 5:** Proportional subdistribution hazards models for breast cancer synchronous brain metastases.

**Variables**	**Categories**	**Univariable**		**Multivariable**	
		**sHR (95%CI)**	***p*-Value**	**sHR (95%CI)**	***p*-Value**
Age	Continuous	1.004 (0.997, 1.009)	0.226	1.007 (1.000, 1.013)	0.049
Year	2011	Ref.	.	Ref.	-
	2012	0.834 (0.665, 1.047)	0.117	0.792 (0.628, 0.999)	0.049
	2013	0.868 (0.700, 1.077)	0.198	0.804 (0.648, 0.998)	0.047
	2014	0.819 (0.654, 1.026)	0.083	0.753 (0.596, 0.951)	0.017
	2015	0.715 (0.560, 0.915)	0.008	0.634 (0.491, 0.818)	< 0.001
	2016	0.732 (0.525, 1.019)	0.064	0.664 (0.473, 0.933)	0.018
Race	NHW	Ref.		Ref.	
	NHB	1.247 (1.035, 1.503)	0.02	1.082 (0.877, 1.335)	0.464
	NHAI/AN	0.843 (0.430, 1.655)	0.62	1.122 (0.621, 2.028)	0.702
	NHAPI	0.881 (0.657, 1.180)	0.396	0.976 (0.716, 1.332)	0.88
	Hispanic	0.852 (0.677, 1.072)	0.173	0.856 (0.669, 1.094)	0.214
Region	Northeast	Ref.		Ref.	
	Midwest	1.131 (0.833, 1.537)	0.43	0.889 (0.633, 1.248)	0.495
	South	1.089 (0.877, 1.350)	0.44	0.822 (0.623, 1.084)	0.165
	West	0.881 (0.719, 1.079)	0.221	0.831 (0.663, 1.042)	0.108
Marital status	Married	Ref.		Ref.	
	Others	1.316 (1.137, 1.525)	<0.001	1.191 (1.016, 1.397)	0.031
Insurance status	Insured	Ref.		Ref.	
	Others	1.267 (1.089, 1.475)	0.002	1.221 (1.034, 1.442)	0.018
Primary tumor site	Central	Ref.		Ref.	
	Upper-inner	1.252 (0.809, 1.940)	0.313	1.226 (0.771, 1.952)	0.389
	Lower-inner	0.589 (0.320, 1.083)	0.089	0.689 (0.364, 1.306)	0.254
	Upper-outer	1.103 (0.772, 1.575)	0.591	1.195 (0.810, 1.763)	0.369
	Lower-outer	1.126 (0.691, 1.836)	0.634	1.337 (0.806, 2.217)	0.26
	Axillary tail	0.514 (0.187, 1.413)	0.197	0.555 (0.188, 1.639)	0.286
	Others	1.175 (0.843, 1.638)	0.34	1.183 (0.821, 1.704)	0.367
T-Stage	1	Ref.		Ref.	
	2	1.227 (0.934, 1.610)	0.141	1.270 (0.961, 1.678)	0.093
	3	1.445 (1.081, 1.930)	0.013	1.438 (1.057, 1.956)	0.021
	4	1.689 (1.321, 2.160)	<0.001	1.612 (1.242, 2.091)	<0.001
	Others	1.244 (0.950, 1.627)	0.112	1.235 (0.929, 1.642)	0.146
LNPRate	0–20%	Ref.		Ref.	
	21–40%	0.846 (0.432, 1.657)	0.626	0.893 (0.455, 1.750)	0.741
	41–60%	0.824 (0.414, 1.642)	0.583	0.997 (0.441, 2.255)	0.994
	61–80%	0.991 (0.448, 2.193)	0.982	0.923 (0.442, 1.927)	0.83
	81–100%	0.894 (0.549, 1.456)	0.653	0.815 (0.508, 1.308)	0.397
	Unexamined	1.023 (0.656, 1.595)	0.919	0.962 (0.626, 1.478)	0.861
	Others	0.998 (0.624, 1.595)	0.993	1.031 (0.654, 1.625)	0.894
Subtype	HR+/HER2–	Ref.		Ref.	
	HR+/HER2+	0.950 (0.764, 1.181)	0.643	0.949 (0.755, 1.193)	0.655
	HR–/HER2+	1.030 (0.813, 1.305)	0.804	0.954 (0.744, 1.222)	0.708
	Triple-negative	1.581 (1.299, 1.924)	<0.001	1.693 (1.374, 2.086)	<0.001
	Unknown	1.117 (0.887, 1.406)	0.346	1.135 (0.890, 1.447)	0.308
Bone metastases	No	Ref.		Ref.	
	Yes	1.023 (0.879, 1.191)	0.771	1.001 (0.848, 1.181)	0.994
Liver metastases	No	Ref.		Ref.	
	Yes	1.361 (1.170, 1.583)	<0.001	1.367 (1.163, 1.607)	<0.001
Lung metastases	No	Ref.		Ref.	
	Yes	1.242 (1.075, 1.435)	0.003	1.114 (0.957, 1.298)	0.163
Income*	Continuous	0.367 (0.245, 0.552)	<0.001	0.320 (0.169, 0.608)	0.001
Education*	Continuous	1.010 (0.998, 1.021)	0.113	0.998 (0.982, 1.014)	0.767

Income^*^, median household income, increased by per $10 000 annual; Education^*^, high school education percent, increased by per 10%; NHW, Non-Hispanic White; NHB, Non-Hispanic Black; NHAI/AN, Non-Hispanic American Indian/Alaska Native; NHAPI, Non-Hispanic Asian or Pacific Islander.

## Discussion

In a large population-based retrospective cohort study, we examined the complicated association between age and the presence and survival of BCSBMs. As far as we know, this is an epidemiologic study with great interest and novelty based on the SEER program that will generally offer a superior understanding of variation in the onset and prognosis of BCSBMs. The presence of BCSBMs was shown to have a U-shaped relationship with age, with the maximum HR occurring at the age of 62. The association between age and all-cause and cause-specific mortality, on the other hand, resembled linear behavior.

### Comparison with other studies

In recent years, there has been an increasing amount of literature on the epidemiology of BMs at the diagnosis of systemic malignancy (12–14, 30). Using data from the SEER database from 2010 to 2013, Martin et al. (13) and Cagney et al. (14) found that 0.41 percent of adult breast cancer patients have synchronous BMs in the United States. In earlier research assessing the 2014–2016 SEER data, we found that 0.42 percent of midlife breast cancer diagnosed in the United States have synchronous BMs. The current study found similar results, and the incidence fraction of BCSBMs remained generally steady from 2011 to 2016.

In reviewing the above-mentioned literature, no solid evidence was found for the association between age and the presence of BMs. As mentioned in the research of Cagney et al. (14) among all patients with cancer, age 41–60 years (vs. age 18–40 y, OR 1.55, 95% CI: 1.39–1.71, *p* < 0.05) was associated with higher risk of having synchronous BMs, whereas age > 80 years (vs. age 18–40 y, OR 0.60, *p* < 0.05) had significantly lower odds and age 61–80 years had non-significantly greater odds (vs. age 18–40 y, OR 1.08, *p* > 0.05). According to another study concentrating on BCSBMs, the relationship between age and the occurrence of synchronous BMs was more elusive and harder to reconcile because OR values of age 41–60 years, 61–80 years, and > 80 years (vs. age 18–40 years) were all non-applicable (13). We reason that these seemingly misleading results might be due to the use of age as a categorical variable in the adjusted logistic regression model. Naturally, we assumed that treating age as a continuous variable would help to ameliorate the situation and lead to a positive conclusion. In this case, however, age (increased by per 1, OR 1, 95% CI: 0.99–1.00, *p* = 0.49) was not related to the presence of BMs (12). Another report discovered that age ≤ 40 years (vs. age > 40 years, HR 2.10, 95% CI 1.02–4.36) was associated with an increased risk of developing metachronous BMs in HER2-positive breast cancer (31). Additional research showed no information on the effect of age on the occurrence of BCSBMs (11, 32, 33).

A possible explanation for this might be that prior research relied on the essential assumption that the presence of BMs was related linearly to age (12–14, 33). For ordinal or continuous factors, the linearity assumption may be inappropriate, and more elaborate interactions may be necessary (34). Conversely, cubic splines are commonly used because they offer a lot of flexibility when it comes to fitting data, are visually smooth due to their continuous first and second derivatives, and have fewer fit constants than higher degree splines (35–39). In this study, we discovered that using a regression spline to solve such situations is a preferable option. Our findings on age were not in line with the previous findings, whereby we observed a U-shaped association (p for non-linearity <0.001) with the presence of BMs after accounting for potential confounders. Further research revealed that the relationships between age and the occurrence of BCSBMs are approximately linear in both the younger (p for non-linearity = 0.163) and older (p for non-linearity = 0.068) age groups. Consequently, we were able to convert a complex non-linear association into a linear one. Undoubtedly, therefore, age may represent a clinical marker for early identification of a population at high risk for having BCSBMs.

The National Comprehensive Cancer Network (NCCN), American Society of Clinical Oncology (ASCO), and European School of Oncology-Metastatic Breast Cancer guidelines for breast cancer do not recommend routine imaging assessment or continued imaging reassessment of BMs for breast cancer because the overall incidence of BMs is relatively low in the general BC population and there is no proven benefit from non-randomized retrospective studies (40–42). Imaging assessment of the brain is recommended only after the appearance of central nervous system symptoms (43, 44). Nonetheless, timely identification of BMs is critical for BC patients since it may allow better therapeutic responses than delays in diagnosis (45, 46). Research finds that the combination of early detection and advanced therapies (both local and systemic) ultimately leads to more favorable outcomes (47). Although routine screening of all BC patients is not justified, screening people who are at high risk might be beneficial.

For all malignancies, age is a well-validated prognosticator related to survival (21, 48, 49). And previous studies have found that being younger at the time of BCSBMs diagnosis is one of the characteristics that predicts a better outcome (12–14, 50, 51). To determine the association between age and the survival of BCSBMs, many researchers used Cox proportional hazards models (13, 22, 52). To our knowledge, none of them has carried out a comprehensive analysis to determine whether a Cox model would be appropriate in this circumstance. Our current study has significant strengths that compensate for the lack of information. Linear associations between age and ASM (p for non-linearity = 0.264) and CSM (p for non-linearity = 0.473) were discovered in this investigation. Those results suggest that the HR and 95%CI of age obtained by Cox models are reasonable.

However, one study found that in multivariate Cox regression analysis, younger age at first diagnosis of BMs in breast cancer patients was a predictor of shorter OS (52). We believe what the authors stated was a mistake after reviewing the original findings in the Supplementary material, which indicated that the risk of all-cause death rose with age (increased by per 1, HR = 1.02, 95% CI 1.01–1.03, *p* < 0.001). Another study found that in a univariable Cox model, age did not correlate with breast cancer BMs survival time (22). A possible explanation for this might be the potentially inappropriate classification of age groups. The research of Martin et al., on the other hand, performs better (13). Martin's study has strength in using Fine and Gray's competing risk regression models to analyze BCSBMs CSM (13). Yet, the study did not include CIF curves or sHR values for each variable. Here, we presented the sHR for the occurrence of CSM and gave plots of all cumulative incidences for categorical variables, as indicated by a prior study (24). Mortality risk tends to increase with age in all regression models, and age-related increases in the risk of CSM are substantially overestimated by the standard Cox model (53).

Age is commonly utilized as a covariate in investigations to build prognostic models for predicting the survival of BC patients with BMs, such as a recursive partitioning analysis (RPA) strategy (54) and graded prognostic assessment (GPA) (55, 56). In the era of individualized therapy, the accurate prediction of BC patients with BMs is critical for optimizing care (18, 57). To some extent, the current study is significant in determining the ability of prognostic tools in future research and an improved prognosis for those individuals.

### Strengths and limitations

The discovery of a U-shaped association between age and the presence of cancer, as well as approximate linear behaviors between age and ASM and CSM, in a large, nationally representative sample of US general cancer patients from the SEER database with rigorous capture of death events, which was undetected by previous excellent work, is a major strength of our study. At the same time, the results of our competing risk model help to compensate for the lack of survival analyses.

Our research, however, has several limitations. First, in the current research, only the presence or absence of BMs at the diagnosis of the study population was provided. Data on whether metastases develop in the brain in the subsequent course of the disease is not available at this time for the SEER program. Our study did not include the data of some patients who later acquired brain metastases, which might have influenced the accuracy of the results (58). Second, the SEER program is a population-based study being carried out primarily in the United States, which has concerns for generalizability outside of the United States (59, 60). Third, we did not report sHR for the competing event, which may have resulted in a bias toward a better understanding of BCSBMs survival (61, 62).

## Conclusion

In conclusion, utilizing a nationally representative database from the United States, this study discovered that age had a non-linear U-shaped relationship with the presence of BCSBMs and a linear relationship with BCSBMs mortality. This article lays the groundwork for future studies. And a better understanding of the complex association might aid in developing age-appropriate public health guidelines.

## Data availability statement

Publicly available datasets were analyzed in this study. This data can be found at: https://seer.cancer.gov.

## Ethics statement

This study was reviewed and approved by the First Affiliated Hospital of Jinan University. Written informed consent for participation was not required for this study in accordance with the national legislation and institutional requirements.

## Author contributions

WC had the initial idea, analyzed the data, and wrote the paper. WC, YW, XW, and JL contributed to study design and commenting on drafts and revisions. All authors contributed to the article and approved the submitted version.

## Conflict of interest

The authors declare that the research was conducted in the absence of any commercial or financial relationships that could be construed as a potential conflict of interest.

## Publisher's note

All claims expressed in this article are solely those of the authors and do not necessarily represent those of their affiliated organizations, or those of the publisher, the editors and the reviewers. Any product that may be evaluated in this article, or claim that may be made by its manufacturer, is not guaranteed or endorsed by the publisher.
