# Supplementary material for: Association between age and the presence and mortality of breast cancer synchronous brain metastases in the United States: A neglected SEER analysis
Source: Front Public Health. 2022 Sep 23;10:1000415. doi: 10.3389/fpubh.2022.1000415 (PMC9539918; doi:10.3389/fpubh.2022.1000415)
Supplement: Supplementary file 1 [file Data_Sheet_1.PDF]

Supplementary Material online

Supplement to: Wenqiang Che, Yujiao Wang, Xiangyu Wang, Lyu Jun. Association between age and presence and mortality of breast cancer synchronous brain metastases in the United States: a neglected SEER analysis

Figure S1.1 Association between age and the presence of breast cancer synchronous brain metastases by race.
Figure S1.2 Association between age and the presence of breast cancer synchronous brain metastases by region.
Figure S1.3 Association between age and the presence of breast cancer synchronous brain metastases by marital status.
Figure S1.4 Association between age and the presence of breast cancer synchronous brain metastases by insurance status.
Figure S1.5 Association between age and the presence of breast cancer synchronous brain metastases by tumor site.
Figure S1.6 Association between age and the presence of breast cancer synchronous brain metastases by T-stage.
Figure S1.7 Association between age and the presence of breast cancer synchronous brain metastases by LNPRate.
Figure S1.8 Association between age and the presence of breast cancer synchronous brain metastases by subtype.
Figure S1.9 Association between age and the presence of breast cancer synchronous brain metastases by bone metastases.
Figure S1.10 Association between age and the presence of breast cancer synchronous brain metastases by liver metastases.
Figure S1.11 Association between age and the presence of breast cancer synchronous brain metastases by lung metastases.
Figure S2. Curves of all cumulative incidences for categorical variables.

Figure S1.1 Association between age and the presence of breast cancer synchronous brain metastases by race.

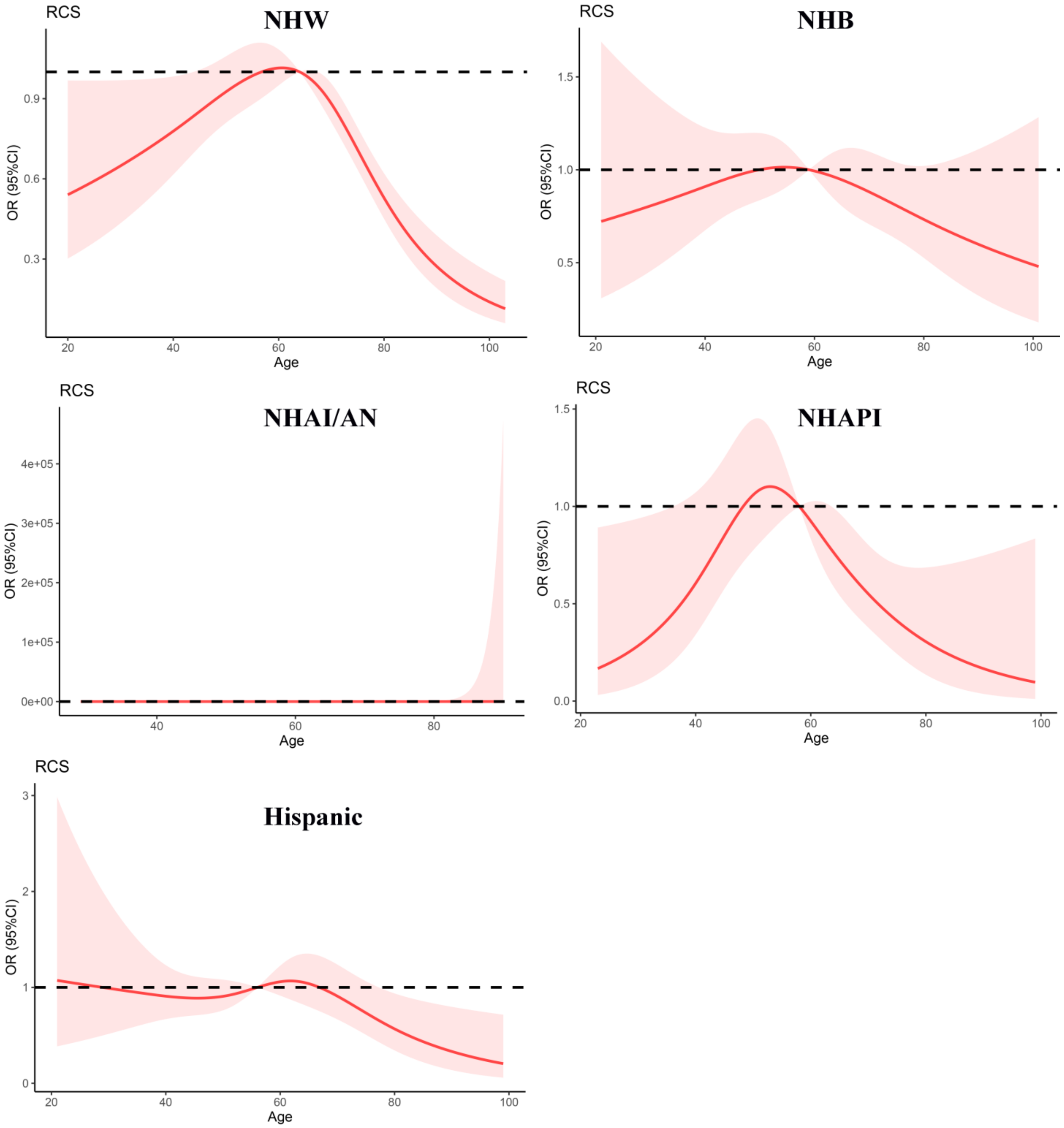

Figure S1.2 Association between age and the presence of breast cancer synchronous brain metastases by region.

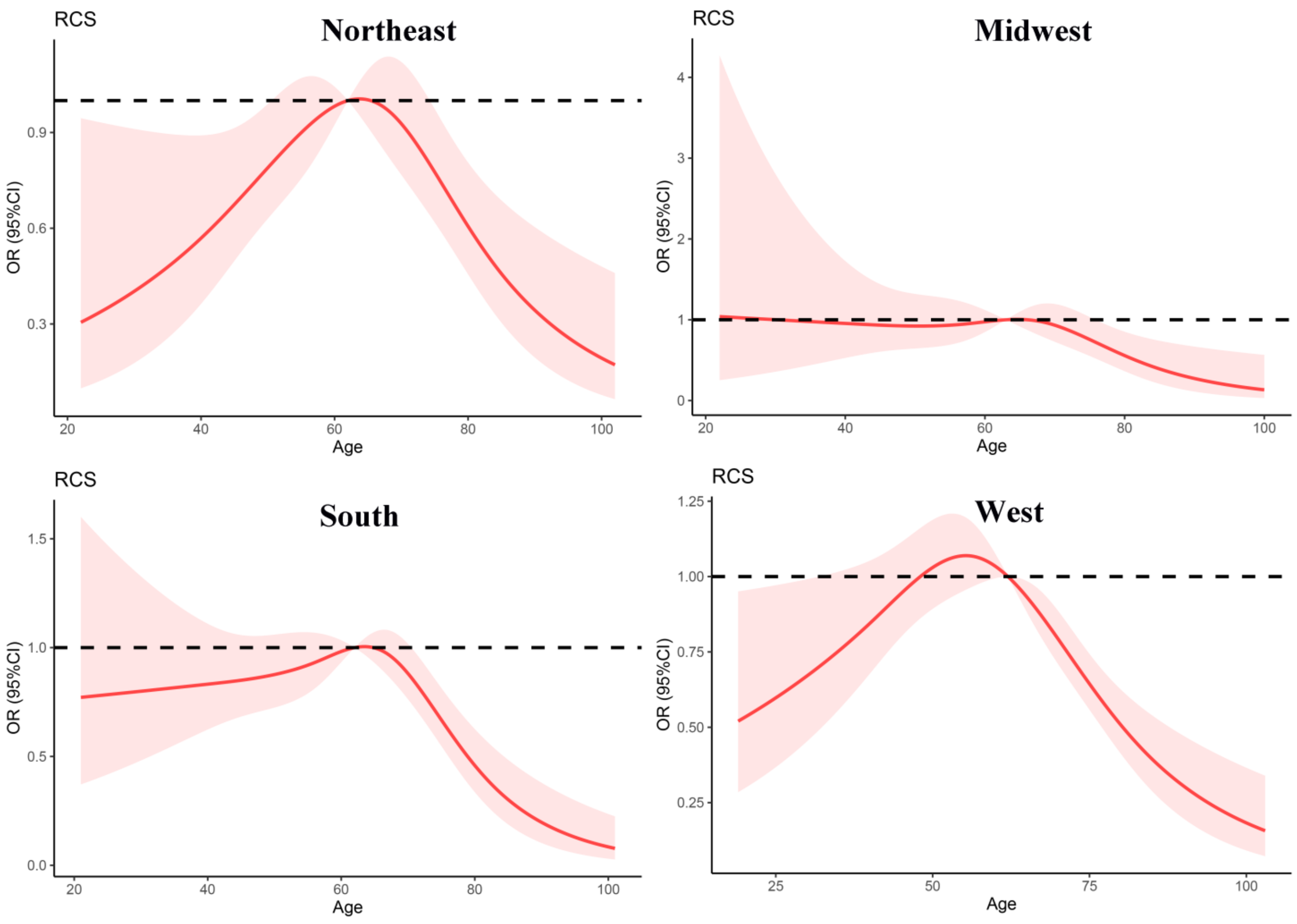

Figure S1.3 Association between age and the presence of breast cancer synchronous brain metastases by marital status.

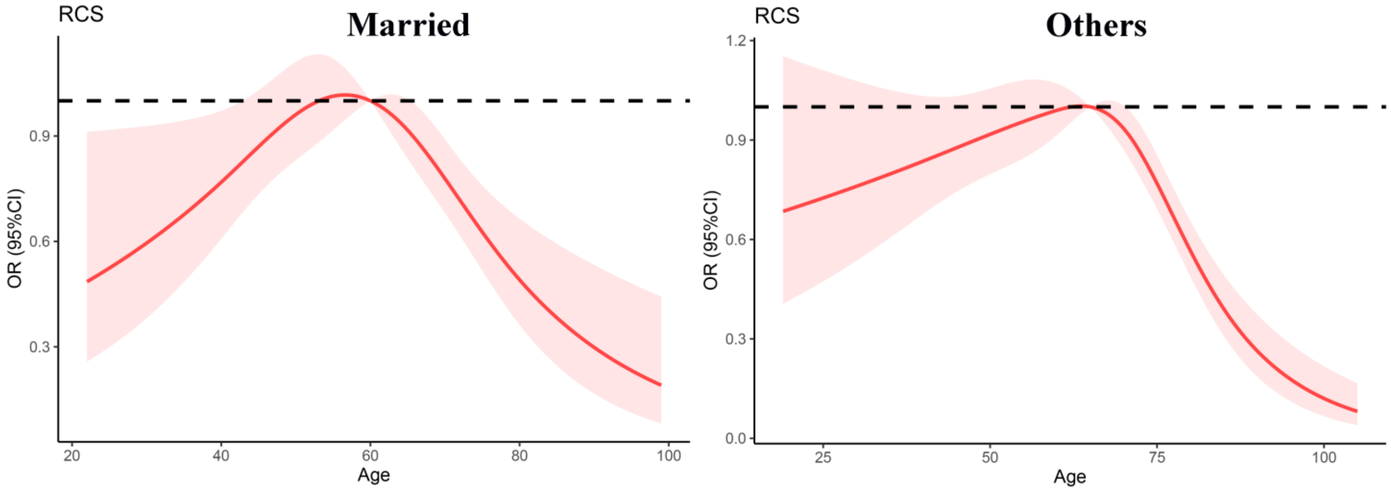

Figure S1.4 Association between age and the presence of breast cancer synchronous brain metastases by insurance status.

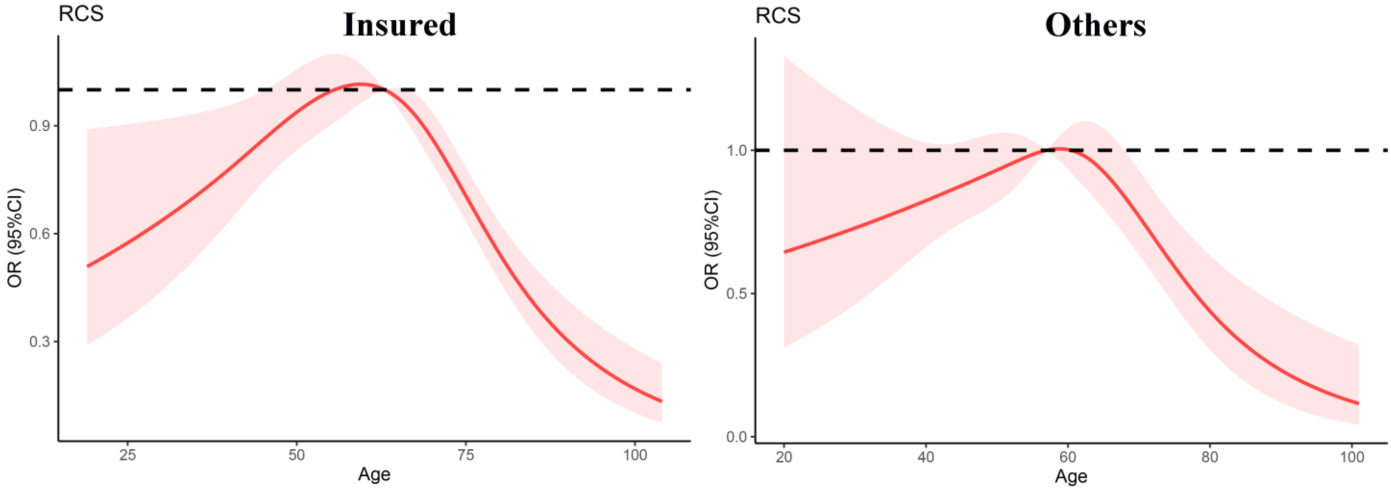

Figure S1.5 Association between age and the presence of breast cancer synchronous brain metastases by tumor site.

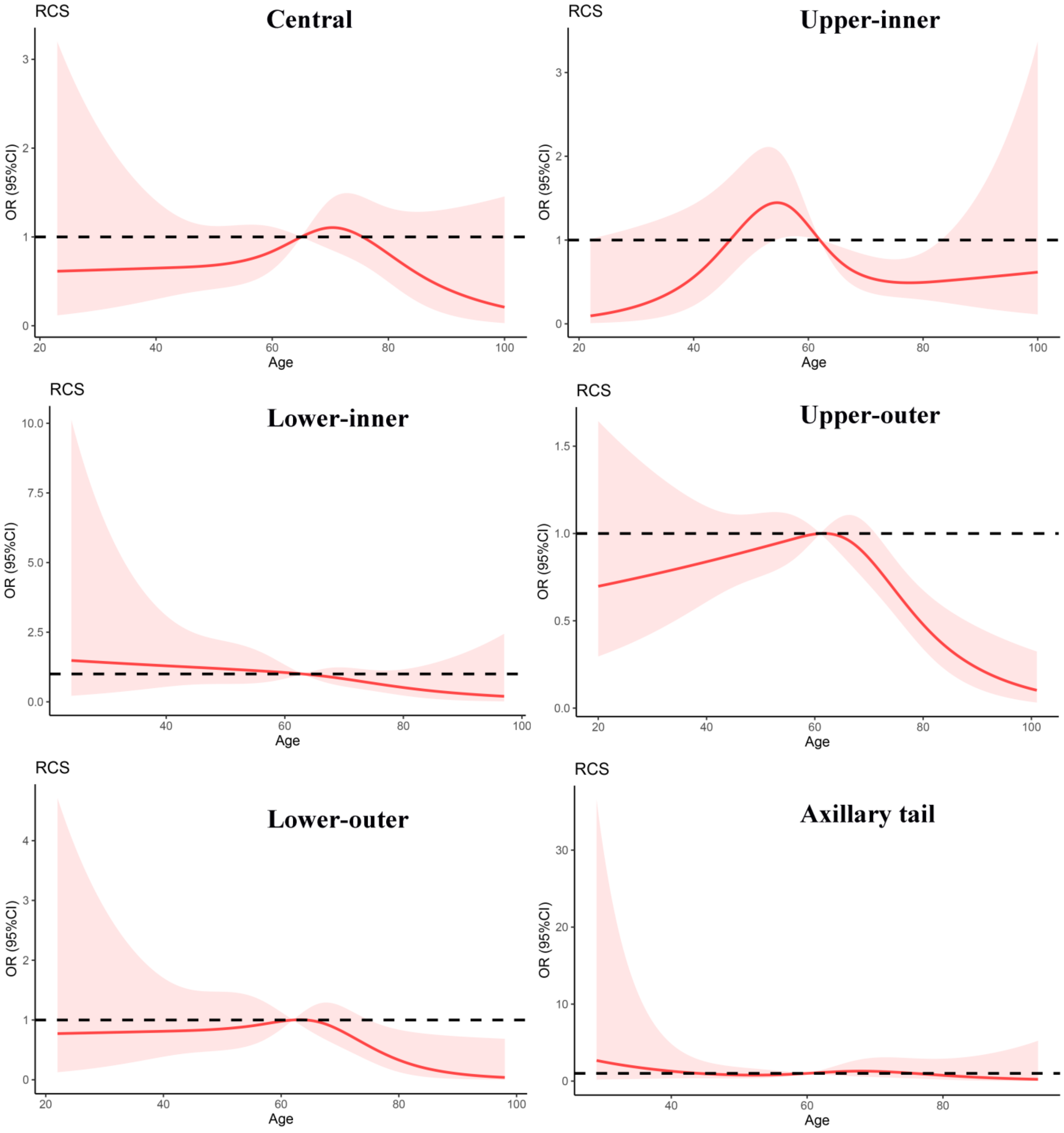

Figure S1.6 Association between age and the presence of breast cancer synchronous brain metastases by T-stage.

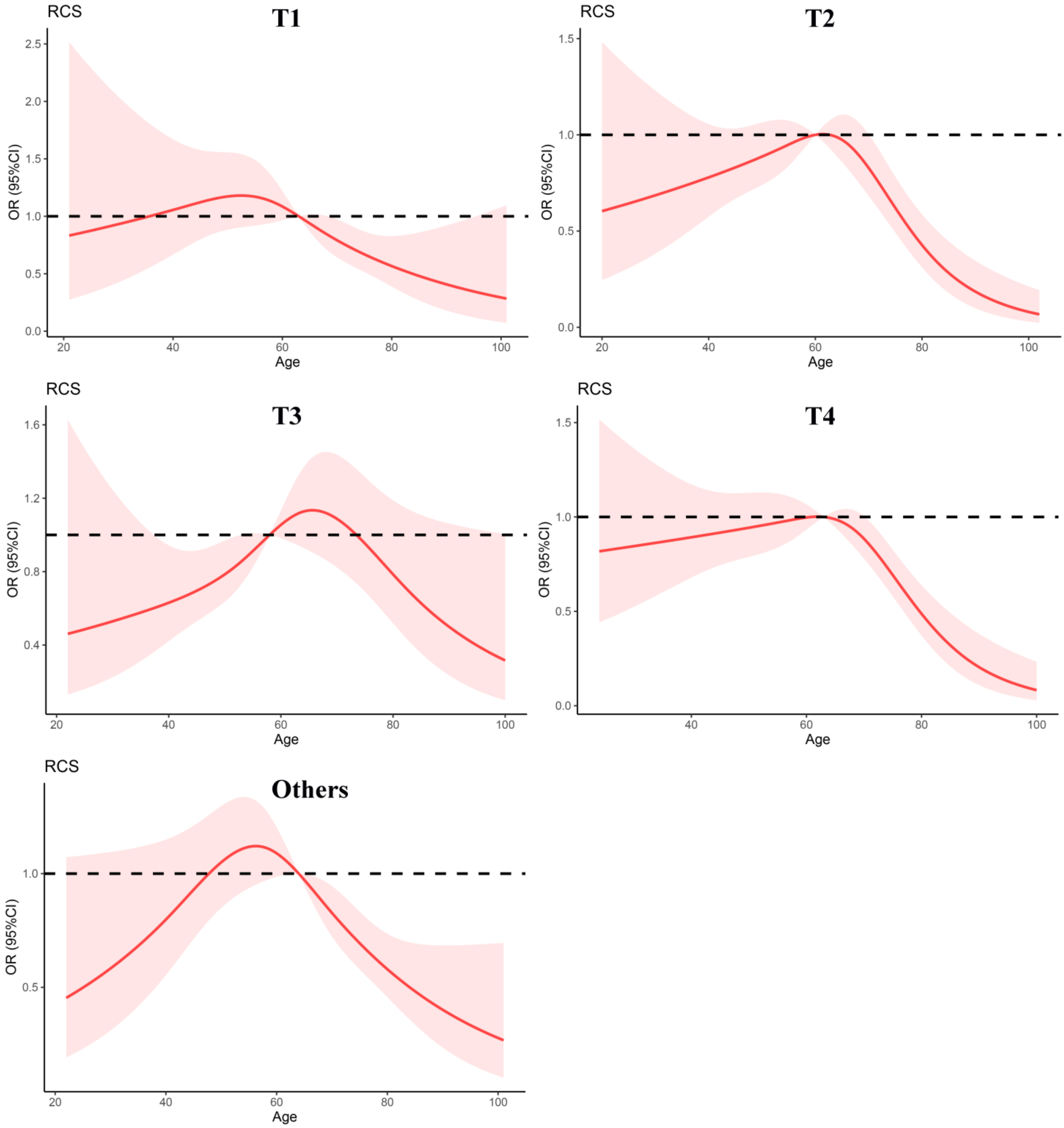

Figure S1.7 Association between age and the presence of breast cancer synchronous brain metastases by LNPRate.

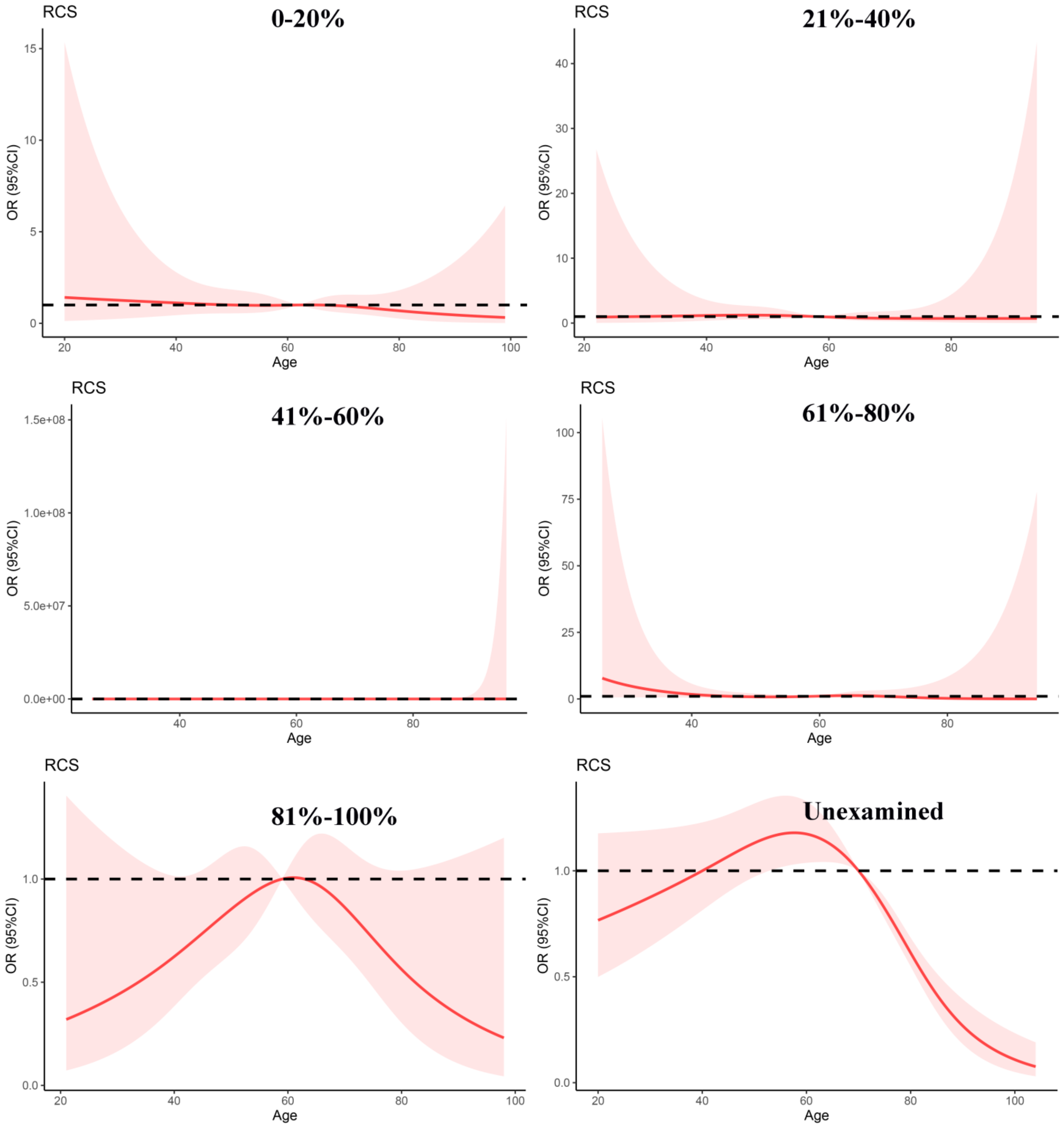

Figure S1.8 Association between age and the presence of breast cancer synchronous brain metastases by subtype.

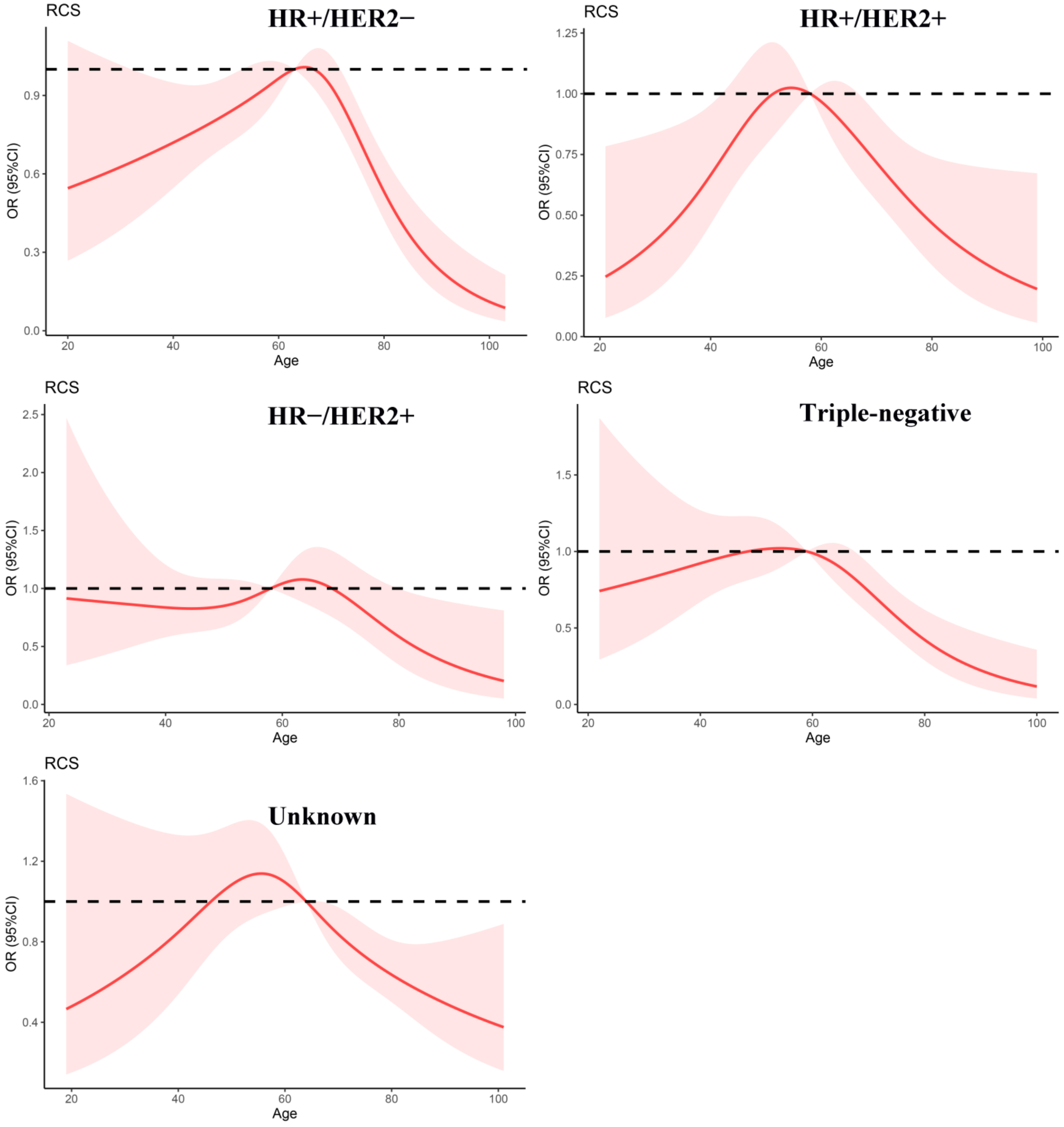

Figure S1.9 Association between age and the presence of breast cancer synchronous brain metastases by bone metastases.

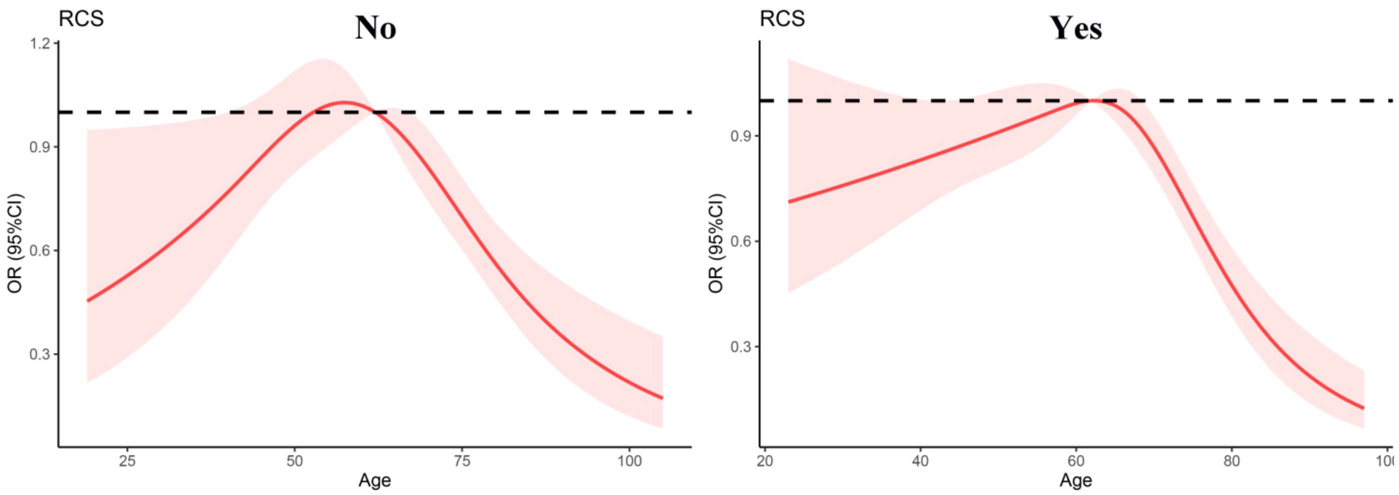

Figure S1.10 Association between age and the presence of breast cancer synchronous brain metastases by liver metastases.

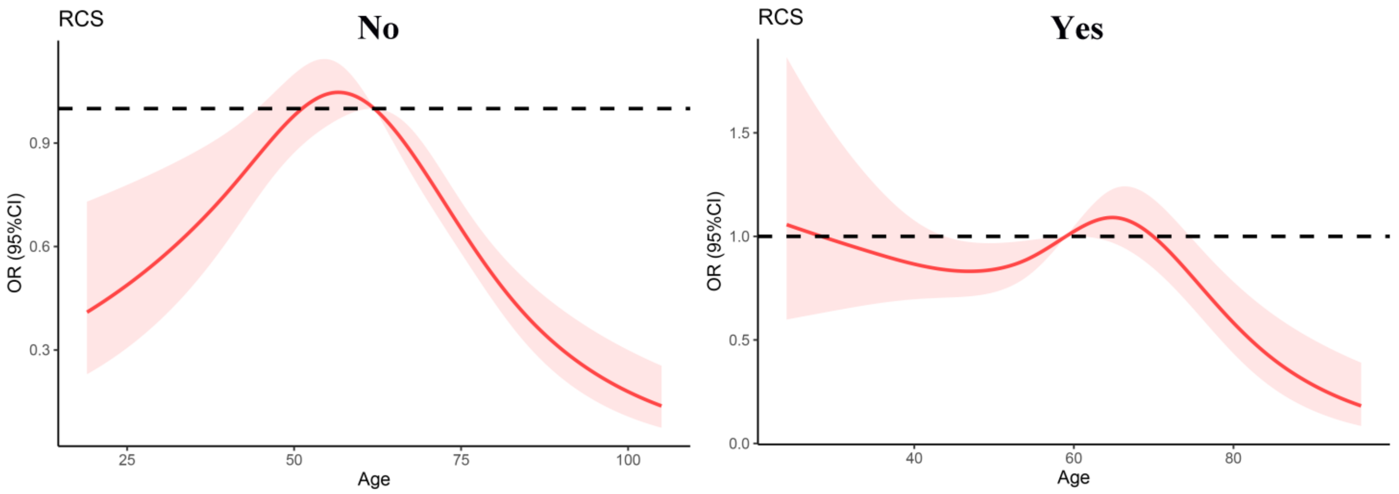

Figure S1.11 Association between age and the presence of breast cancer synchronous brain metastases by lung metastases.

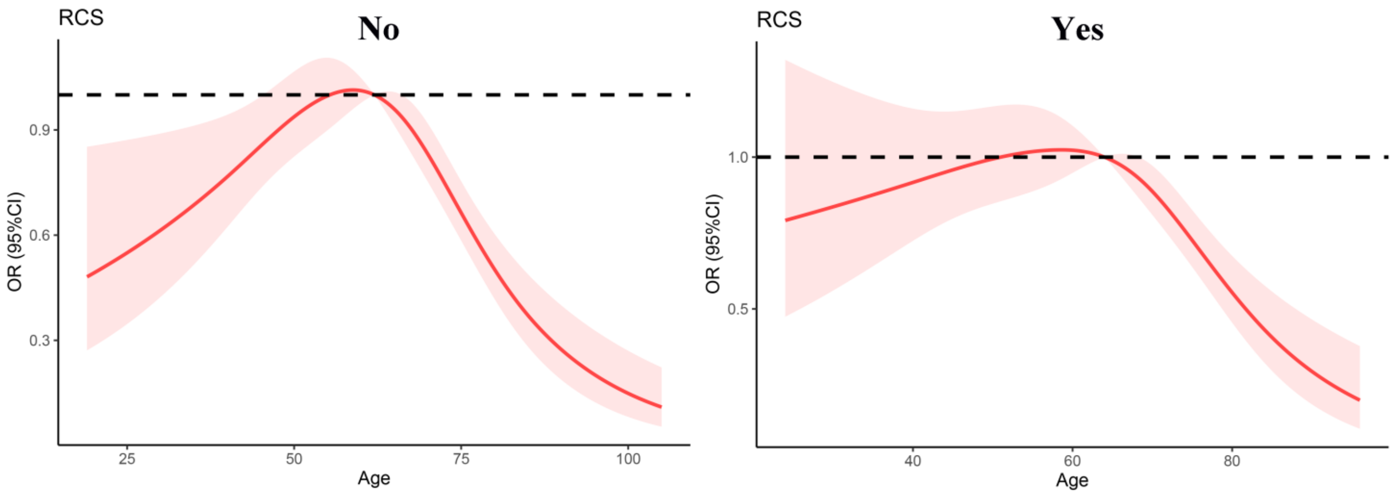

Figure S2. Curves of all cumulative incidences for categorical variables.

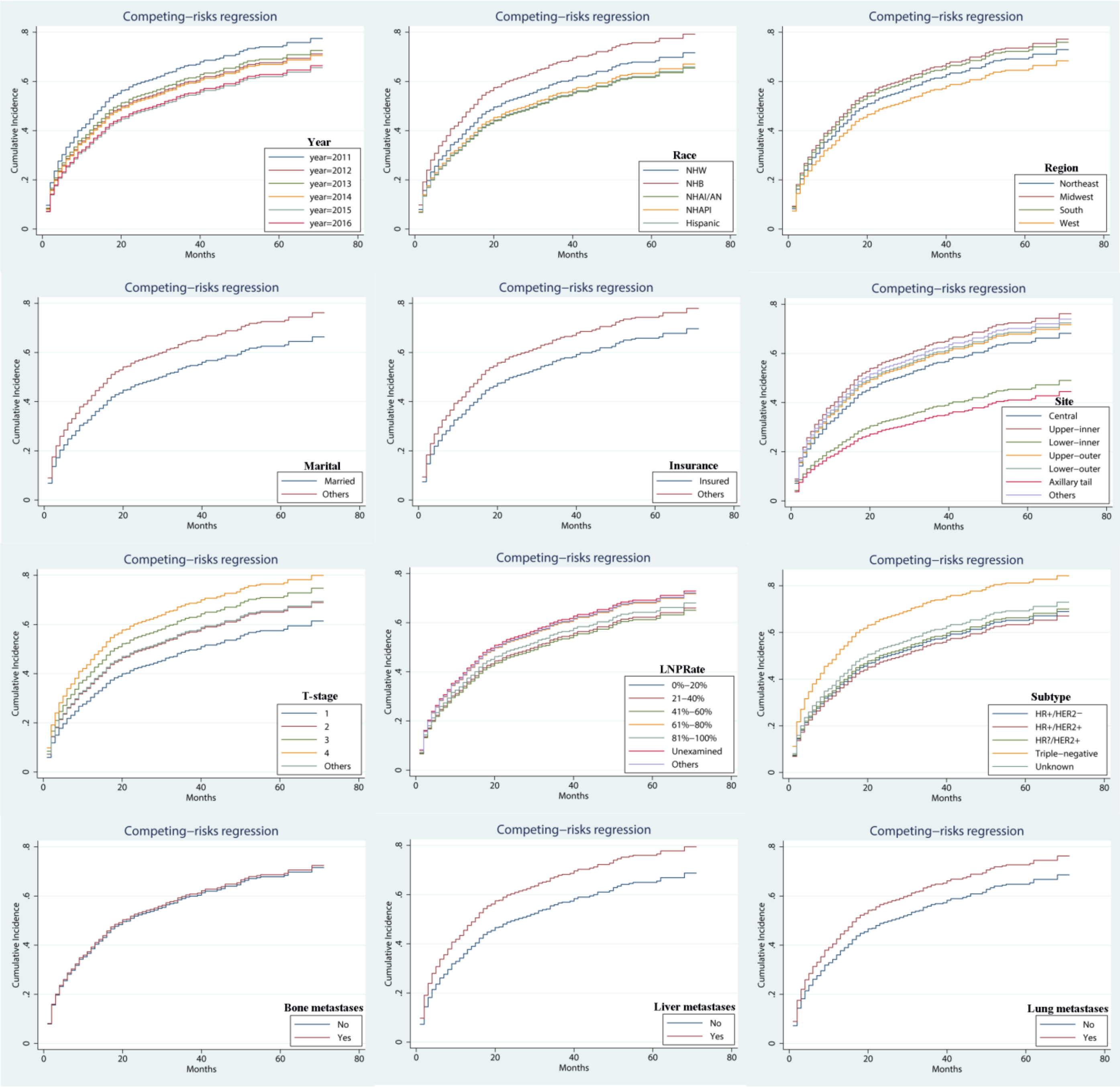

-
